# Simulated lateral tunneling for treating a huge submucosal tumor at the cervical esophagus

**DOI:** 10.1055/a-2277-0375

**Published:** 2024-03-14

**Authors:** Yue Zhao, Huige Wang, Dan Liu

**Affiliations:** 1 191599 Department of Gastroenterology and Hepatology, The First Affiliated Hospital of Zhengzhou University, Zhengzhou, China

**Fig. 1:**
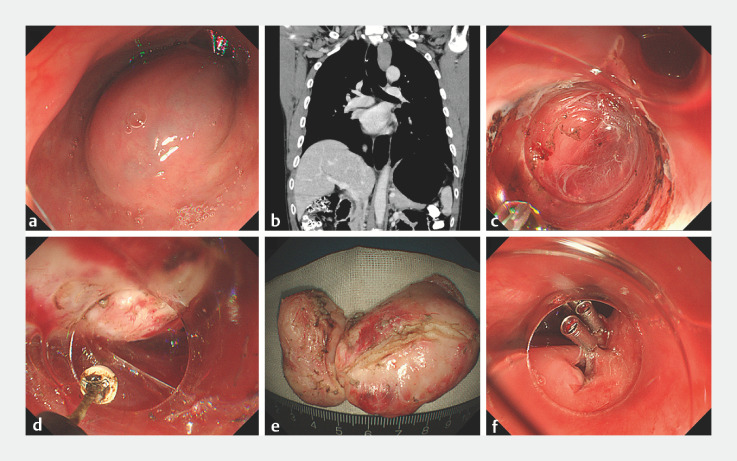
**a** Esophageal submucosal tumor located in the cervical esophagus. **b** Computed tomography scan showed a huge transluminal mass (arrows) adjacent to the introitus esophagus. **c** A longitudinal incision was made along one side of this tumor to create a simulated lateral tunnel in a step-wise manner. **d** The tumor was gradually dissected as deep as the bottom around the lesion using an insulated knife. **e** The resected specimen. **f** The lateral tunnel access was closed by clips.

A 43-year-old man was referred to our hospital for treatment of a huge submucosal tumor adjacent to introitus esophagus identified by surveillance endoscopy ( Fig. 1 **a** ). An enhanced computed tomography demonstrated a marked submucosal tumor measuring 2.6 × 8 cm, protruding intra- and extraluminally ( Fig. 1 **b** ). After multidisciplinary team discussion and sufficient informed consent, endoscopic resection with simulated lateral tunneling was scheduled to remove this mass ( Video 1 ).

We demonstrate a derivative technique of submucosal tunneling endoscopic resection, creating a simulated lateral tunnel to resect a huge submucosal tumor at the cervical esophagus.Video 1

After submucosal injection, a longitudinal incision was made along one side of this tumor in a step-wise manner ( Fig. 1 **c** ). Inside the submucosal tunnel, the tumor was gradually dissected as deep as the bottom around the lesion using an insulated knife ( Fig. 1 **d** ). During the procedure, a snare acted as traction to expose the base of the tumor and facilitate the dissection. Postoperatively, the specimen was retrieved through the mouth ( Fig. 1 **e** ), and the lateral tunnel access was closed by clips uneventfully ( Fig. 1 **f** ). The patient resumed a liquid diet 2 days after the procedure without any adverse events. Histopathology and immunohistochemistry revealed a diagnosis of leiomyoma.

Submucosal tunneling endoscopic resection (STER) has been widely applied for esophageal submucosal tumors with safety and effective advantages. However, the submucosal lesion at the cervical esophagus is difficult to treat by traditional STER, because there is no space to build a procedural plane. In this video, we demonstrate a derivative technique of STER, creating a simulated lateral tunnel to resect a huge submucosal tumor at the cervical esophagus, a technique that has proved to be feasible and safe management.

Endoscopy_UCTN_Code_TTT_1AO_2AG

